# Preservation of gait biomechanics during offloading treatment of diabetic foot ulcers

**DOI:** 10.1186/1757-1146-7-S1-A26

**Published:** 2014-04-08

**Authors:** Claudia Giacomozzi

**Affiliations:** 1 Department of Technology and Health, Istituto Superiore di Sanità, Rome, Italy

## Background

Gold standard for the management of diabetic foot neuropathic ulcers is the irremovable total contact cast, however evidence of equal clinical effectiveness was proved for the removable walker OPTIMA DIAB [1]. Priority of this healing treatment is the offloading of the ulcerated foot area; however, the healing process may last for months, and eventual changes in gait biomechanics may entail risks for the contralateral foot, leg joints, or even spine. Aim of this study is to setup a reliable methodology to optimize the use of such offloading devices.

## Materials and methods

The OPTIMA DIAB (Molliter, Italy) is a special boot with an external rigid rocker sole, a fiber-glass rigid interface, a multi-layer modular insole, and a posterior rigid brace. To investigate forces at the boot external and internal interface, a Kistler force platform and an in-shoe Pedar baropodometer were used. The assessment protocol was applied to a healthy volunteer (F; 41years; BMI 19; high forefoot pressures; negligible foot extra-rotation; used to walk fast): 10 consistent footsteps per foot were simultaneously acquired by the two systems when the subject hit the platform; a high cadence of 110 steps per minute was acoustically imposed; the subject was acquired while walking barefoot (B), with primary prevention flexible shoes (F), with secondary prevention rigid shoes (R), with the OPTIMA DIAB without offloading (O), and with the OPTIMA DIAB with right central forefoot offloading (OS1). Pressure footprints were divided into 4 major regions [2]. All relevant parameters were averaged, with force and COP curves resampled before averaging.

## Results

**Figure 1:**
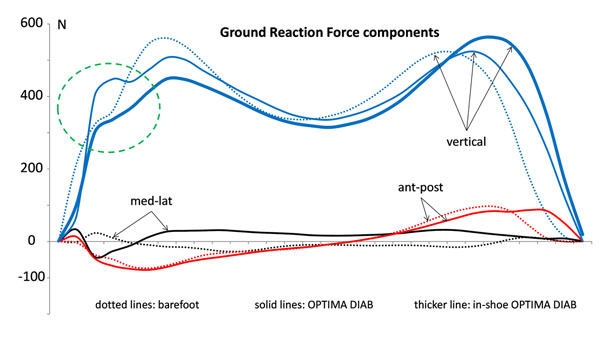
Mean GRF curves of B (dotted lines) and O (solid lines) conditions, measured by the force platform, and mean vertical force of O (thicker blu line) condition, obtained from the in-shoe pressure system. All curves have been averaged over 10 consistent trials.

**Figure 2:**
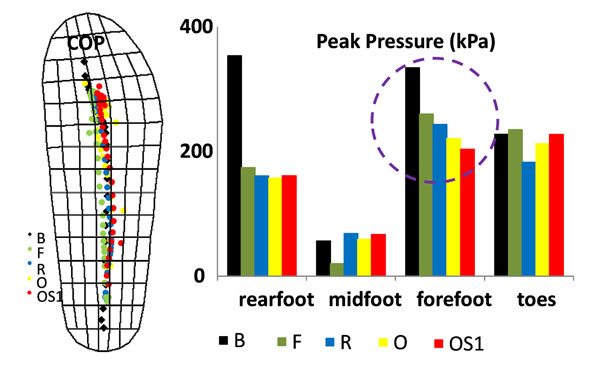
Mean COP trajectories under B, F, R, O and OS1 conditions; mean peak pressures under the same conditions and associated with rearfoot, midfoot, forefoot and toes. Curves and values have been averaged over 10 consistent trials.

The OPTIMA DIAB force curves were 3% delayed in propulsion with respect to all other conditions. A significant change was also found at heel strike (Figure 1), entailing alterations in COM acceleration and body instantaneous adaptation. In-shoe force confirmed the modified impact at the foot interface, even though partly smoothed. Prevention solutions F, R O and OS1 were increasingly effective in reducing forefoot pressure (B: 334.2±30.3kPa; F: 260.0±17.3kPa; R: 243.3±29.3kPa; O: 220.8±19.8kPa; OS1: 203.5±17.0kPa); comparison between O and OS1 in the specific offloaded area showed lower peak pressure (88.3±6.9kPa, -34%), pressure-time integral (21.3±2.2kPa×s, -49%), stance duration (0.41±0.06s, -20%). However, differences were found in timing and loading pattern of all regions, as well as in COP excursion (Figure 2). Differences and asymmetries between R and O (or OS1) conditions are worth of special attention since they are often used in combination, i.e. the contralateral foot wearing a rigid sole prevention shoe.

## Conclusions

The used methodology seem to be valuable, also in a clinical setting, for the analysis of biomechanical changes induced by the offloading device. The hereby highlighted alterations, although magnified by the purposely high cadence and the rigid surface of the force platform, should be taken into account when designing the treatment. Further investigation is in progress to find out indicators to optimize the intervention, i.e. to effectively offload injured areas while preserving reasonable loading pattern and gait symmetry.
